# Splenic Infarction Induced by Dengue Hemorrhagic Fever: A Rare Presentation

**DOI:** 10.7759/cureus.17072

**Published:** 2021-08-10

**Authors:** Waleed Hafiz, Faisal Alotaibi, Raghad Alneefia, Elaf Alghuraibi, Abdulaziz Basha Ahmed, Ashraf Warsi

**Affiliations:** 1 Internal Medicine • Rheumatology, Umm Al-Qura University, Mecca, SAU; 2 Medicine, Al Noor Specialist Hospital, Mecca, SAU; 3 Internal Medicine, Umm Al-Qura University, Mecca, SAU; 4 Internal Medicine, Jazan University, Jazan, SAU; 5 Internal Medicine, Al Noor Specialist Hospital, Mecca, SAU

**Keywords:** splenic infarction, dengue fever, dengue hemorrhagic fever, hematological manifestations, vascular manifestations

## Abstract

Haematological and vascular features of dengue virus infection are common and vary from tiny skin haemorrhages to significant bleeding such as epistaxis, gastrointestinal bleeding and hematuria. Spontaneous splenic rupture has also been reported as an atypical manifestation in dengue fever. We report a case of splenic infarction in a 35-year-old man who presented with fever, vomiting, diffuse abdominal pain and distention, diarrhoea, hematuria, headache, back pain, hypotension, pleural effusion and ascites. Laboratory evaluation confirmed the diagnosis of dengue hemorrhagic fever, and abdominal imaging revealed splenic infarction. He required intensive care, responded well to inotropic support and remarkably improved.

## Introduction

Apart from its well-known manifestations of plasma leakage leading to circulatory failure, dengue hemorrhagic fever (DHF) can result in multiple other complications, such as encephalopathy, myocarditis, hepatitis and haematological and vascular manifestations. It can also affect the spleen and lead to spontaneous splenic rupture, splenic hematoma and splenomegaly [1]. Splenic infarction usually occurs when septic or bland emboli occlude the splenic artery or one of its branches. Other causes for splenic infarction include blood-borne malignancies, abdominal trauma, pancreatic disorders, hyper-coagulable states, autoimmune diseases, vascular disorders and infections [2]. The literature review reveals several reported cases of infections that were complicated with splenic infarction. These include Epstein-Barr virus, cytomegalovirus, malaria and brucellosis [3-5]. In this report, we present a case of splenic infarction associated with DHF.

## Case presentation

A 35-year-old man with no past medical or surgical history, presented with a two-day history of persistent and high-grade fever. This was associated with chills, myalgia, vomiting, diffuse abdominal pain, back pain and headache. He denied contact with sick individuals, recent travel, or possibly raw or contaminated food or milk consumption. There were no similar episodes in family members, friends or co-workers. Over the counter analgesics did not provide any relief, and thus, he decided to seek medical advice. Upon presentation to the emergency room, his temperature was 38.8 degrees Celsius, and his heart rate was 112 beats per minute. His other vital signs were normal. The tourniquet test was positive. He had evidence of scleral jaundice. Abdominal examination revealed diffuse abdominal tenderness without organomegaly. The rest of his physical examination was unremarkable. Initial laboratory panel revealed the following: white blood cell count 3.9x10^9/L, haemoglobin 13g/L, platelet count 22x10^9/L, international normalized ratio (INR) 1.08, prothrombin time (PT) 12, partial thromboplastin time (PTT) 49, fibrinogen level 2.2g/L, aspartate transaminase (AST) 289u/L, alanine transaminase (ALT) 184u/L, albumin 28g/L, total bilirubin 24μmol/L, direct bilirubin 20μmol/L, urea 3.28mmol/L and creatinine 122μmol/L. Peripheral blood smear showed red blood cells with mild hypochromia, microcytosis and anisocytosis, mild leucocytosis with shifting to the left, toxic granulation, and absolute lymphocytosis, few basket cells and marked thrombocytopenia with normal morphology. There was no evidence of malaria on a peripheral blood smear. 

He was admitted to the medical ward as a possible cause of acute viral illness with associated severe thrombocytopenia and hepatitis. Symptomatic treatment and broad-spectrum antibiotics were initiated with close monitoring of his platelet count and liver transaminases. Further workup was requested, including serologic testing for dengue virus, cytomegalovirus, hepatitis A, B and C viruses and brucella. Blood and urine cultures were also requested. 

**Figure 1:**
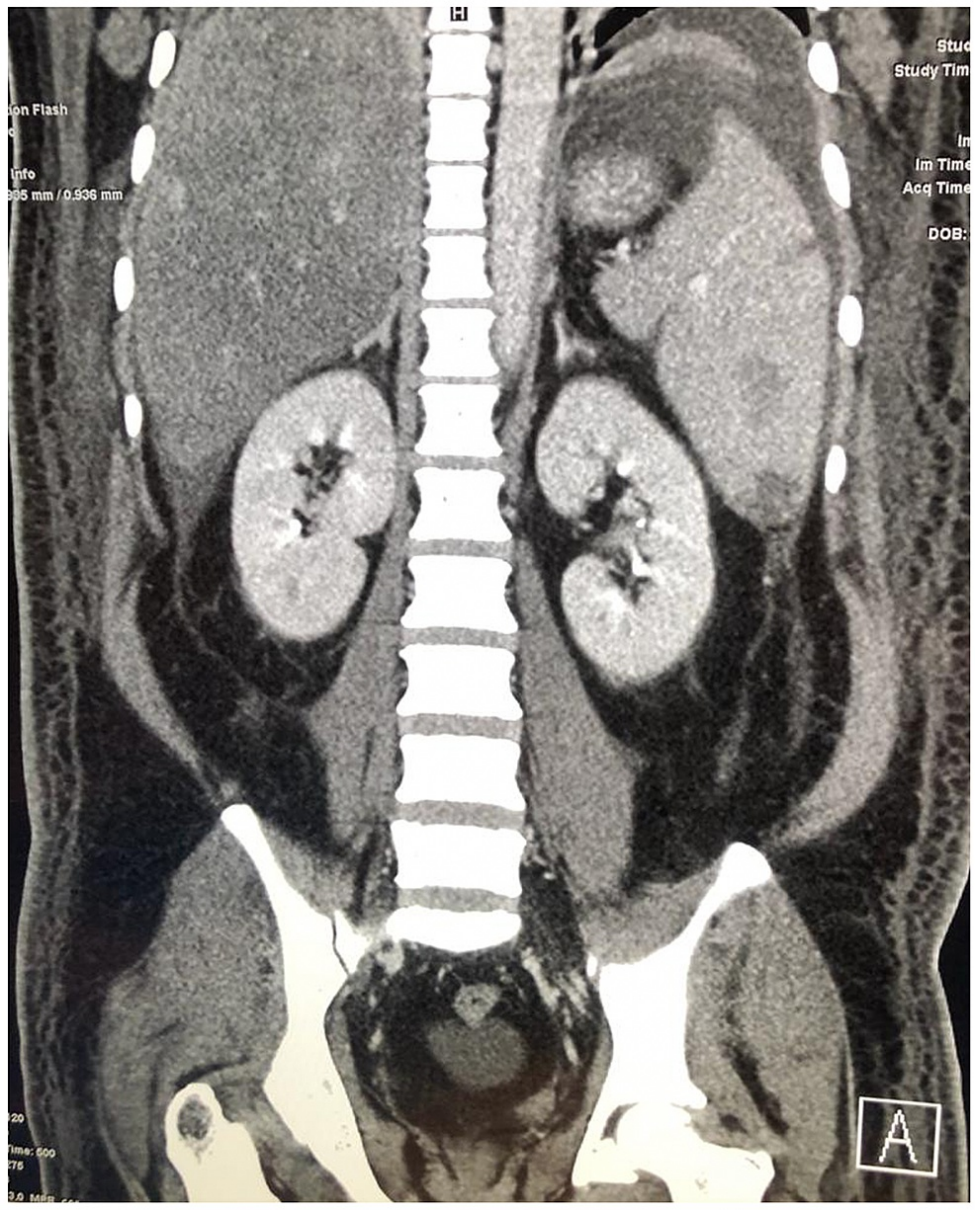
Abdominal CT shows multiple hypodensities across the spleen.

**Figure 2:**
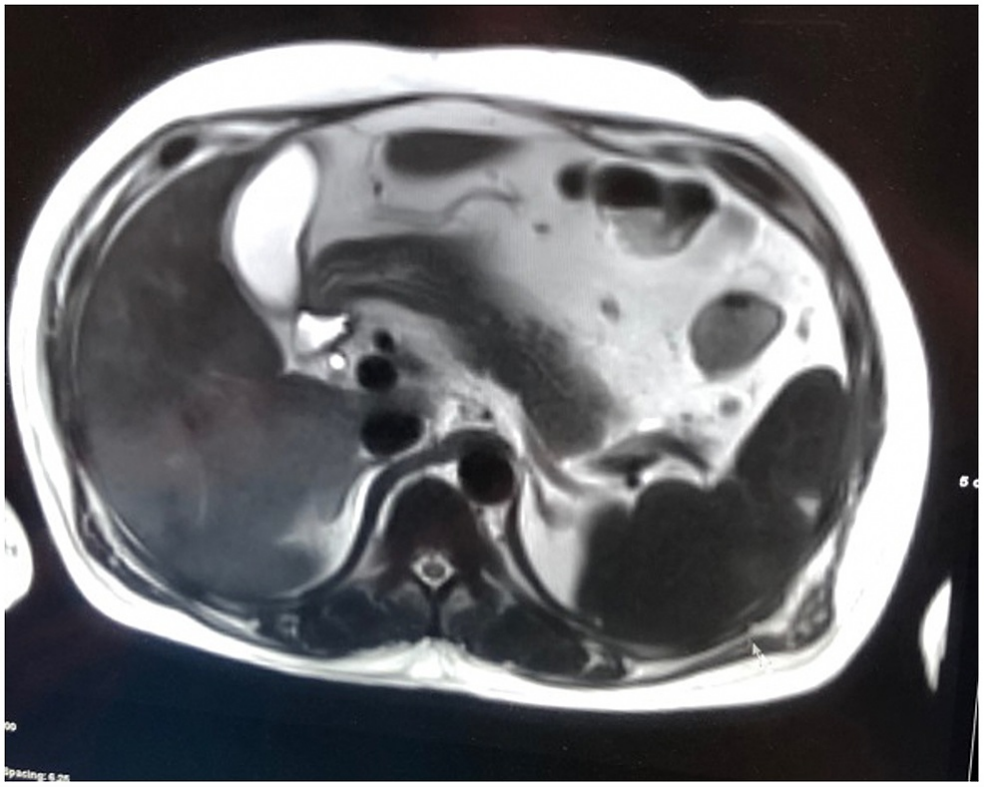
MRI of the abdomen reveals peripheral wedge-shaped defects with decreased signal intensity in the spleen, confirming the diagnosis of splenic infarction.

On the next day of admission, his condition started to worsen. He developed generalized anasarca and shortness of breath. His abdominal pain becomes more intense with new-onset distention. Physical examination revealed hypotension, bilateral basal lung crepitations, moderate ascites and bilateral lower limb edema. Electrocardiogram, cardiac enzymes, D-dimer and echocardiography, were all normal. Chest X-ray showed bilateral pleural effusion, and abdominal ultrasound demonstrated moderate ascites. He did not respond to intravenous fluid therapy and was shifted to the intensive care unit. Abdominal computed tomography (CT) showed a moderate amount of ascitic fluid in the abdomen, fatty liver infiltration and multiple irregular areas of hypodensity in the spleen with no enhancement on intravenous contrast and otherwise normal splenic size (figure 1). Magnetic resonance imaging (MRI) of the abdomen confirmed the diagnosis of splenic infarctions (figure 2). During his stay in the intensive care unit, results of the pending workup were reported. Serology for dengue virus tested positive in the form of high titers of immunoglobulin M (IgM) and immunoglobulin G (IgG) and positive dengue virus polymerase chain reaction (PCR). The rest of the viral serologies and blood and urine culture were all negative.

Based on that, the diagnosis of splenic infarction associated with DHF was confirmed. His circulatory failure was treated with inotropic support and symptomatic management. After about three weeks of intensive care, his clinical condition remarkably improved, and his laboratory parameters normalized (Table 1). He was then safely discharged home.

**Table 1:** The course of some of the selected laboratory parameters for the patient throughout his admission period. PTT- partial thromboplastin time; INR- International normalized ratio; PT- prothrombin time; ALT- Alanine transaminase; AST- Aspartate transaminase;

Laboratory test	Day 1	Day 3	Day 7	Day 10	Day 15	Day 20	Day 25
Hemoglobin (g/L)	13.9	14.6	12.5	10.7	11.7	9.3	7.91
Leucocytes (10^9/L)	3.9	18.7	8.9	7.4	9.3	9.3	8.4
Platelets )10^9/L)	22	12	45	92	192	178	607
PTT (seconds)	49	23.2	35.5	39.6	44.2	38.1	79
INR	1.08	1.01	1.29	1.68	1.41	1.45	1.31
PT (seconds)	12.9	12	15.5	19.2	16.9	17.3	15.7
ALT (u/L)	184	435	1777	623	161	103	77
AST (u/L)	289	1168	3402	2145	516	303	168
Total bilirubin (μmol/L)	24	34	57	52	50	40	35
Lactate dehydrogenase (u/L)	-	8091	3031	1350	739	-	327

## Discussion

Despite being a common febrile viral illness with numerous haematological and vascular manifestations, these manifestations in the setting of dengue fever have rarely been reported. Up to our knowledge, this is the first case report of splenic infarction associated with DHF.

Dengue fever is a mosquito-borne viral infection caused by a single positive-stranded RNA virus of the family Flaviviridae, genus Flavivirus. This virus is transmitted to humans through bites of female Aedes aegypti mosquito [6,7]. It affects individuals of all age groups. Clinical presentation varies from subclinical or mild infection to severe symptoms like DHF that might progress to dengue shock syndrome (DSS) and eventually death [8].

Individuals with DHF present with sudden onset of fever, which usually lasts for up to 7 days, and several nonspecific symptoms and signs. The most frequently noted hemorrhagic features are skin petechiae, ecchymoses and purpuric lesions. Gingival bleeding, gastrointestinal haemorrhage, epistaxis and hematuria are less frequently reported. Patients can also test positive for the tourniquet test, which reflects increased capillary fragility and may help diagnose. Distinguishing DHF from dengue fever and other viral infections is difficult during the acute stage of the illness. However, hemorrhagic complications and possibly circulatory collapse may follow within 24 to 48 hours from the onset of symptoms [6-9]. 

Leukopenia and thrombocytopenia are common in DHF. Hemoconcentration as a result of plasma leakage is noted in almost all cases of classic DHF. Elevated liver enzyme levels are common. Laboratory criteria for the diagnosis of dengue fever include one or more of the following: Demonstration of dengue virus antigen in serum samples via enzyme immunoassay (NSI-ELISA), detection of viral genomic sequences in serum via reverse-transcriptase polymerase chain reaction (RT-PCR), demonstration of a fourfold or greater change in reciprocal IgG or IgM antibody titers to one or more dengue virus antigens or isolation of dengue virus from serum, plasma or leukocytes [7-9].

Similar to several viral illnesses, patients with dengue fever usually improve spontaneously. They only require symptomatic management and supportive measures such as analgesics, rest and fluid replacement. To date, no antiviral treatment has been approved as a specific treatment for dengue fever. Management of DHF should focus on fluid replacement and prevention and treatment of haemorrhage [10]. Methylprednisolone has failed to reduce mortality in patients with dengue shock syndrome [11]. 

In Saudi Arabia, dengue fever is endemic in the Western Province. The two Holy Cities of Makkah and Madinah are destinations of pilgrims who assemble from all over the world every year to perform Hajj and Umrah. Furthermore, urbanization, international travelling and the fast-growing trade in the province have led to several reported outbreaks of dengue fever over the past two decades [12]. Splenic infarction is a condition in which arterial or venous blood flow to the spleen is compromised, leading to subsequent infarction, tissue necrosis and ischemia. It can affect a segment of the spleen or lead to global splenic infarction [13].

Patients with splenic infarction present with a wide clinical spectrum ranging from being asymptomatic to fatal haemorrhage. The classic symptom is severe left upper quadrant abdominal pain. This pain is associated occasionally with fever, chills, nausea and vomiting. Laboratory evaluation is performed to exclude another differential diagnosis. Radiological assessment is crucial to establish the diagnosis. The abdominal CT scan and MRI are the imaging modalities of choice, although ultrasound abdomen is an alternative option. Management of splenic infractions requires treatment of the underlying etiology and providing adequate pain relief. Splenectomy is reserved for complicated clinical situations [14]. 

The exact mechanism of splenic infarction remains unclear, yet several patho-etiologies were proposed in the literature. These include hyper-coagulable states, induction of inflammation, tissue damage secondary to infectious process [15], direct endothelial damage, septic embolism, blunt abdominal trauma [16], post-liver transplant and Pancreatectomy [17]. 

Our patient proposes that induction of the pro-inflammatory response secondary to endothelial cell activation and endothelial injury seems to be the most favourable theoretical explanation as there was no evidence of sepsis or hypercoagulopathy.

## Conclusions

Splenic infarction is a rare but life-threatening complication of DHF. Clinical suspicion should rise in a patient presenting with left upper quadrant pain in the setting of dengue fever. The patho-etiology of splenic infarction in DHF is interesting yet not explored. We advocate for the theory that endothelial injury and induction of inflammation may lead to splenic infarction. We think that more cases of splenic infarction in DHF will help further explore this association.

## References

[1] de Silva WT, Gunasekera M (2015). Spontaneous splenic rupture during the recovery phase of dengue fever. BMC Res Notes.

[2] Chapman J, Helm TA, Kahwaji CI (2021). Splenic Infarcts. https://www.ncbi.nlm.nih.gov/books/NBK430902/.

[3] Wand O, Tayer-Shifman OE, Khoury S, Hershko AY (2018). A practical approach to infarction of the spleen as a rare manifestation of multiple common diseases. Ann Med.

[4] Hwang JH, Lee CS (2014). Malaria-induced splenic infarction. Am J Trop Med Hyg.

[5] Alyousef M, Enani M, Elkhatim M (2015). Acute brucellosis with splenic infarcts: a case report from a tertiary care hospital in Saudi Arabia. Case Rep Infect Dis.

[6] Brady OJ, Gething PW, Bhatt S (2012). Refining the global spatial limits of dengue virus transmission by evidence-based consensus. PLoS Negl Trop Dis.

[7] Fakeeh M, Zaki AM (2001). Virologic and serologic surveillance for dengue fever in Jeddah, Saudi Arabia, 1994-1999. Am J Trop Med Hyg.

[8] Sharma SK, Kadhiravan T (2008). Spontaneous splenic rupture in dengue hemorrhagic fever. Am J Trop Med Hyg.

[9] Miranda LE, Miranda SJ, Rolland M (2003). Case report: spontaneous rupture of the spleen due to dengue fever. Braz J Infect Dis.

[10] Hasan S, Jamdar SF, Alalowi M, Al Ageel Al Beaiji SM (2016). Dengue virus: a global human threat: review of literature. J Int Soc Prev Community Dent.

[11] Tassniyom S, Vasanawathana S, Chirawatkul A ( 1993). Failure of high-dose methylprednisolone in established dengue shock syndrome: a placebo-controlled, double-blind study. Pediatrics.

[12] Alhaeli A, Bahkali S, Ali A, Househ MS, El-Metwally AA (2016). The epidemiology of dengue fever in Saudi Arabia: a systematic review. J Infect Public Health.

[13] Schattner A, Adi M, Kitroser E, Klepfish A (2015). Acute splenic infarction at an academic general hospital over 10 Years: presentation, etiology, and outcome. Medicine (Baltimore).

[14] Bottomley MJ, Gibson M, Alchi B (2019). PR3 vasculitis presenting with symptomatic splenic and renal infarction: a case report and literature review. BMC Nephrol.

[15] La Russa VF, Innis BL (1995). 11 mechanisms of dengue virus-induced bone marrow suppression. Baillieres Clin Haematol.

[16] Cabello-Gutiérrez C, Manjarrez-Zavala ME, Huerta-Zepeda A (2009). Modification of the cytoprotective protein C pathway during dengue virus infection of human endothelial vascular cells. Thromb Haemost.

[17] Wu SC, Chen RJ, Yang AD, Tung CC, Lee KH (2008). Complications associated with embolization in the treatment of blunt splenic injury. World J Surg.

